# Liver Resection for Colorectal Secondaries

**DOI:** 10.1155/1992/89252

**Published:** 1992

**Authors:** Paul H. Sugarbaker

**Affiliations:** The Cancer Institute Washington Hospital Center 110 Irving Street, NW Washington DC 20010 USA


*HPB Surgery*, 1992, Vol. 6, pp. 65–68
Reprints available directly from the publisher
Photocopying permitted by license only

© 1992 Harwood Academic Publishers GmbH
Printed in the United Kingdom

**HPB INTERNATIONAL**

**EDITORIAL & ABSTRACTING SERVICE**

JOHN TERBLANCHE, EDITOR
Department of Surgery,
Medical School · Observatory 7925
Cape Town · South Africa
Telephone: (021) 47-1250 Ext. 2
Telefax (021) 448-6461

### LIVER RESECTION FOR COLORECTAL SECONDARIES

### ABSTRACT

*Scheele, J., Stangl, R., Altendorf-Hofmann, A. and Gall, F.P. (1991) Indicators of prognosis after hepatic resection for colorectal secondaries. Surgery;* **110**: 13–29.

**From 1960 to 1988, 266 patients underwent resection of colorectal secondaries to the liver with curative intent. All patients were followed until April 1, 1990, or death, with a median follow-up time of 52 months. Nine patients with minimal macroscopic residual disease and 38 patients with all gross tumor removed but positive margins showed a poor prognosis with a median survival time of 13.3 months, the longest being 42 months. Of the 219 patients having potentially curative resection, 12 patients died postoperatively (5.5%). Acturial 5, 10 and 20-year survival for the remaining 207 patients was 39%, 28%, and 18%, respectively. At April 1, 1990, 77 patients were alive with no evidence of disease for up to 24 years, and 12 patients had died without recurrence. The following factors were associated with less favorable crude survival: presence and extent of mesenteric lymph node involvement ($p = 0.0003$), grade III/IV primary tumor ($p = 0.035$), synchronous diagnosis of metastases ($p = 0.017$), satellite metastases ($p = 0.0003$), limited resection margins ($p = 0.019$), and nonanatomic procedures ($p = 0.013$). With respect to disease-free survival, grading of the primary ($p = 0.055$) and the extent of clear margins ($p = 0.19$) failed to achieve statistical significance. Two other criteria are commonly recommended as absolute contraindications to hepatic resection: extrahepatic disease and the presence of four or more independent metastases. A radical excision of all detectable disease may rarely be possible in these circumstances. Nevertheless, within the curative settings, no significant predictive value regarding either overall or disease-free survival was found in this series. Three corresponding "high risk" patients are alive without disease at 5 to 11 years from hepatic resection. These**

**patients with more advanced intrahepatic or concomitant limited extrahepatic disease require a particularly thorough diagnostic work up. As no superior therapeutic alternative is currently available, an aggressive surgical approach may occasionally be justified, and may, in a small portion, result in definite tumor control. (Surgery 1991; 110: 13–29).**

### PAPER DISCUSSION

KEY WORDS: Liver metastases, colorectal carcinoma, liver resection

These authors are to be congratulated on the high quality of their clinical contribution. They present to us a convincing confirmation of some "Indicators of prognosis after hepatic resection for colorectal secondaries" that have been previously described[1]. They add some information previously unavailable. The value of a large single institution study becomes clear as this paper is studied. These authors confirm that positive margins of resection, lymph node invasion present in the primary tumor, and synchronous versus metachronous diagnosis are important prognostic features that must be considered in the selection of patients for resection of liver secondaries.

New information not previously available concerns their work with satellite metastases. Disease free survival and survival were both significantly diminished in the presence of satellite lesions (p value 0.003 and 0.0003 respectively). Apparently, satellite tumor nodules indicate a tumor biology in which the metastatic process is prominent. Local regional metastases in and around the hepatic tumor indicate that systemic metastases are more likely to occur or that other distant cancer foci within the liver are more likely to occur.

However, perhaps that most important clinical message from Erlangen concerns our overall approach to patients with metastatic colorectal cancer. It seems that large bowel cancer as a metastatic process behaves differently than, for example, breast malignancy[2]. Using the terminology of Weiss, *et al.*, colorectal cancer seems to be relatively more metastatically inefficient than breast cancer[3]. Is there a single patient with metastatic breast cancer to lung, liver, or other sites in whom the surgical removal of this disease led to a long term disease free survival?

Dr Scheele and colleagues lead us to conclude that the number one criteria for selection of patients for liver resection or liver resection plus resection of extra hepatic disease is one's ability to remove all clinically evident disease with a clear margin. Contradictory to previously accepted general surgical rules, extra hepatic disease completely resected did not impact adversely on either survival or disease free survival in this group of 266 patients! Both groups of patients had a projected 10 year survival disease free of approximately 30%. The time to recurrence was slightly increased in those patients who had liver metastases plus extra hepatic disease completely resected; but long term follow-up showed that the "surgical complete response" is associated with a respectable long term disease free survival.

Although these data provided by Dr Scheele and colleagues strongly supports the use of surgery in patients with hepatic secondaries, these data do not make the surgeon's task easier. The surgeon's skills must provide: (1) Proper patient selection so that all patients are clinically disease free at the close of an operative procedure; and (2) Sufficient technical expertise to achieve a negative margin of excision, prevent excessive intraoperative blood loss, leave the abdomen hemostatically intact and without bile leaks, and preserve sufficient liver parenchyma as required for an event free postoperative course.

Our studies suggest that the CT portogram may show preoperatively better than any other radiologic study which patients will be left with a positive margin of resection on a major vascular structure and which patients can be resected with a clear margin of normal liver[4]. Others, have made these judgements intraoperatively through the use of an intraoperative ultrasound. Because the CT portogram may prevent unnecessary exploratory surgery in some patients, we have preferred this examination and supplement these findings with intraoperative ultrasound.

Liver surgery as practiced in Erlangen is not for the uninitiated generalist. To achieve the extensive resections performed by this group with a low (5.5%) operative mortality requires special expertise. Also, segment oriented resections to remove multiple metastases and yet preserve parenchyma thereby assuring a smooth postoperative course are technically demanding. Especially those segmental resections that remove liver segments above the transverse plane of the liver (segments, 4A, 8, and 7) should not be attempted without special training and experience as a liver resectionist.

Especially timely is the data presented concerning follow-up surgery. Thirty-four patients with initially curative liver resections underwent reoperation for cancer relapse with curative intent. Eighteen of these patients were again surgical complete responses. At the time of publication, seven of these 18 patients remain alive with no evidence of disease. Again, this information increases rather then relieves the surgeon's burden for giving care to patients with cancer. This means that careful follow-up of patients who have had resection of liver secondaries is required because approximately 10% will recur with resectable disease. This makes them a candidate for further surgery and approximately one quarter of these patients can be salvaged by repeat resections of metastatic disease!

One may ask where does this surgical approach to the treatment of metastatic disease lead us. Can surgery interrupt the cascade phenomenon and change the natural history of a stepwise process into a curable local-regional process? Is there a window-of-time through which surgery can salvage a proportion of large bowel cancer patients from otherwise inevitable death from cancer? What are the implications of these hypotheses for the overall management of colorectal cancer patients? There are physician costs, patient costs, and quality of life implications that must be addressed. Yet, even in our current state of ignorance, I am convinced that the surgical complete response in properly selected patients is durable (ideally 100%) and that it results in cure in $\frac{1}{3}$ of these patients. I feel compelled to continue to offer liver resection or liver resection plus the complete removal of extra hepatic disease to patients who can be made clinically disease free.

Pursuit of a surgical complete response may be a much more reasonable treatment alternative than systemic chemotherapy. Unfortunately, at the present time a majority of patients with liver metastases go straight to chemotherapy treatments and the more promising treatment strategy, surgery for metastatic disease, is ignored until chemotherapy can no longer be employed because of disease progression or drug toxicity. My personal impression is that this unnecessary delay closes the window of surgical opportunity for many patients.

What is needed is groups of people who work together to determine if complete responses with surgery combined with modern chemotherapy in a prospective study will improve the long term survival of these patients treatments. Scheele and colleagues must be congratulated in that they have shown the benefits of a surgical complete response in continued efforts to optimize the surgical care of patients with colorectal cancer. They have also contributed to our fund of knowledge concerning this disease process.

### REFERENCES

1. Hughes, K.S., Simon, R., Sugarbaker, P.H. *et al.* from the Hepatic Metastases Registry (1986) Resection of the liver for colorectal carcinoma metastases: a multi-institutional study of patterns of recurrence. *Surgery*, **100**, 278–284
2. Sugarbaker, P.H. (1989) The natural history of large bowel cancer, surgical implications. *J. Exp. Clin. Cancer Res.*, **8**, (3), 41–43
3. Weiss, L. (1989) Metastatic inefficiency and regional therapy for liver metastases from colorectal carcinoma. *Reg. Cancer Treat.*, **2**, 77–81
4. Sugarbaker, P.H., Nelson, R.C., Murray, D.R., Chezmar, J.L., Bernadino, M. (1990) Liver computerized tomography for hepatic resection: A segmental approach. *Surg. Gynecol. Obstet.*, **171**, 189–195

Paul H. Sugarbaker
Medical Director
The Cancer Institute
Washington Hospital Center
110 Irving Street, NW
Washington, DC 20010
United States of America

